# Pentraxin 3: A Novel Biomarker for Inflammatory Cardiovascular Disease

**DOI:** 10.1155/2012/657025

**Published:** 2012-01-04

**Authors:** Kenji Inoue, Tatsuhiko Kodama, Hiroyuki Daida

**Affiliations:** ^1^Department of Cardiology, Juntendo University Nerima Hospital, Tokyo 177-0033, Japan; ^2^RCAST, LSBM, The University of Tokyo, Tokyo 153-0041, Japan; ^3^Department of Cardiology, Juntendo University School of Medicine, Tokyo 113-0033, Japan

## Abstract

Numerous studies have recently examined the role of pentraxin 3 (PTX3) in clinical situations. The pentraxin family includes C-reactive protein (CRP); however, unlike CRP, PTX3 is expressed predominantly in atherosclerotic lesions that involve macrophages, neutrophils, dendritic cells, or smooth muscle cells. Interestingly, PTX3 gene expression in human endothelial cells is suppressed to a greater extent by pitavastatin than the expression of 6,000 other human genes that have been examined, suggesting that PTX3 may be a novel biomarker for inflammatory cardiovascular disease. The expression and involvement of PTX3 in cardiovascular diseases are discussed in this paper, along with the characteristics of PTX3 that make it a suitable biomarker; namely, that the physiological concentration is known and it is independent of other risk factors. The results discussed in this paper suggest that further investigations into the potential novel use of PTX3 as a biomarker for inflammatory cardiovascular disease should be undertaken.

## 1. Introduction

Biomarkers are measurable and quantifiable biological parameters that can have an important impact on clinical situations. Ideal biomarkers are those that are associated with disease clinical endpoints in observational studies and clinical trials, and in some cases, they may even be used as surrogate endpoints. Biomarkers must also be both independent of established risk factors and recognized to be a factor in the disease for which they are a marker. The normal physiological expression of a potential biomarker must also be known in order to interpret results, as well as to generalize results to various population groups. Finally, potential biomarkers must also have the ability to improve overall prediction beyond that of traditional risk factors, while assays to detect them must have an acceptable cost and be subject to standardization in order to control for the variability of measurements [1].

Basic research over the past decades has identified numerous candidate genes and proteins as biomarkers for cardiovascular disease. In the cardiovascular field, such biomarkers are useful not only for diagnosis but also as indicators of disease trait (risk factor or risk marker), disease state (preclinical or clinical), or disease rate (progression or prognosis) [2]. One protein that has the potential to be a viable biomarker for inflammatory vascular disease is pentraxin 3 (PTX3).

## 2. Pentraxin 3

PTX3 is an evolutionarily conserved, multimeric acute phase inflammatory glycoprotein in the same family as the well-established cardiovascular biomarker C-reactive protein (CRP) [3, 4]. PTX3 also shares 98% identity with tumor necrosis factor- (TNF-) stimulated gene 14 (TSG14) [5, 6]. PTX3 has been successfully identified by Breviario et al. using differential screening of a cDNA library from human umbilical vein endothelial cells (HUVECs) stimulated by interleukin-1 beta [5], as well as by Gustin et al. using the 2D-DIGE approach to detect PTX3 in HUVECs stimulated by lysophospholipids [7]. Our group also identified PTX3 when we were investigating statin as a target gene in HUVECs incubated with pitavastatin for 24 hours prior to RNA extraction [8]. Interestingly, chip analysis has demonstrated that, of the 6,000 human genes that have been investigated for response to pitavastatin treatment, PTX3 gene expression is suppressed in human endothelial cells to the greatest extent.

PTX3 synthesis is stimulated in endothelial cells, macrophages, myeloid cells, and dendritic cells by cytokines and endotoxins such as bacterial products, interleukin-1, and TNF [9–11]. The role of PTX3 in neutrophils has also been gradually elucidated by a number of studies. Once synthesized, PTX3 is predominantly organized into covalent octamers through disulfide bonds [12]. Although PTX3 is mainly localized in lactoferrin positive-specific granules [13, 14], it is translocated to the surface of late apoptotic neutrophils upon stimulation, where it accumulates in blebs and is rapidly released. PTX3 then binds with the high-affinity complement component C1q to initiate the classical pathway of complement activation and facilitate pathogen recognition by macrophages.

## 3. Suitability of PTX3 as a Biomarker

### 3.1. PTX3 Expression in Cardiovascular Diseases

#### 3.1.1. Acute Coronary Syndrome (ACS)

The expression of PTX3 has been found to be increased in patients with acute myocardial infarction (AMI). For instance, Peri et al. observed that patients (*n* = 37) with AMI who were admitted to the coronary care unit within 3.2 ± 3.2 hours of the onset of symptoms had increased plasma PTX3 over time [15]. In this study, plasma PTX3 levels were found to peak at a median of 7.5 hours after AMI, and to return to normal levels after 3 days. Similarly, in murine models of AMI, PTX3 mRNA is expressed within 4 hours of the ligation of the coronary artery, reaches peak levels after 24 hours, and returns to normal levels 3 days later [16]. We have also found that plasma PTX3 levels are increased in patients (*n* = 16) with unstable angina pectoris (UAP; 6.20 ng/mL) [17]. Such findings have led to the investigation of PTX3 expression levels as a potential prognostic indicator of disease. Matsui et al. found that the expression of more than 3.1 ng/mL of PTX3 in patients with UAP/non-ST-elevation MI (*n* = 204) was predictive of the occurrence of a 6-month cardiac event, including cardiac death, rehospitalization for ACS, and rehospitalization for worsening heart failure [18], while Latini et al. have shown that the expression of more than 10.73 ng/mL of PTX3 predicted 3-month mortality in patients with AMI (*n* = 724) [19].

#### 3.1.2. Congestive Heart Failure

PTX3 has also been implicated as a predictor of adverse clinical outcomes in patients with heart failure (*n* = 196) in a study with a median follow-up period of 655 days and an ejection fraction of less than 50% [20]. In a further study by Matsubara et al. that focused on patients with heart failure with normal ejection fraction (HFNEF), plasma PTX3 levels were also found to be increased (3.26 (2.36–4.35) ng/mL). This was observed even in patients with HFNEF, although B-type natriuretic peptide (BNP) was within normal limits [21].

#### 3.1.3. Sleep Apnea Syndrome

Plasma PTX3 levels have also been suggested to be a good marker for the response to treatment of patients with obstructive sloop apnea (OSA). Kasai et al. demonstrated that not only did patients with OSA (*n* = 50) express higher levels of plasma PTX3 than individuals in an age- and body mass index-matched control group, but also that continuous positive airway pressure (CPAP) therapy led to a significant reduction in plasma PTX3 levels. While high sensitive CRP has previously been suggested to be a highly sensitive candidate biomarker that can reflect the status of patients with OSA, the findings of this study led the authors to conclude that plasma PTX3 levels seem to be a more suitable biomarker to monitor treatment effects in patients with OSA [22].

#### 3.1.4. Heart Valvular Disease

In a study by Naito et al. that investigated PTX3 expression patterns in patients with aortic valve stenosis (AS) or regurgitation (AR), it was found that the expression of plasma PTX3 was significantly increased in patients with AS. Furthermore, PTX3 was found to be expressed predominantly in macrophage cells in the aortic valves of these patients [23].

### 3.2. PTX3 Involvement in Cardiovascular Diseases

Several studies have examined why plasma PTX3 levels are increased in patients with cardiovascular disease, and those that have targeted the PTX3 gene in mice suggest that plasma PTX3 levels may increase in order to confer protection against cardiac tissue damage [16, 24]. For instance, in a model of AMI caused by coronary artery ligation, PTX3-knockout mice showed exacerbated heart damage with a greater no-reflow area and increased inflammatory response, including increased neutrophil infiltration, a decreased number of capillaries, and an increased number of apoptotic cardiomyocytes [16]. This phenotype was reversed by the expression of exogenous PTX3.

PTX3 expression has also been examined using double knockout mice in which PTX3 and apolipoprotein E have been targeted. When gene expression in the aortic arches of these mice was analyzed using gene chip, it was found that several transcription factors involved in intracellular proinflammatory signaling, such as nuclear factor-kappa B and the related proteins Irak1, Fos, Jun, GATA3, GATA4, Egr2, and Egr3, were upregulated after the mice had been fed an atherogenic diet for 16 weeks. The mRNA expression levels of intracellular adhesion molecule, vascular cell adhesion molecule-1, endothelial leukocyte adhesion molecule-1, and platelet/endothelial cell adhesion molecule were also found to be increased in the vascular wall of double knockout mice when compared to those of wild-type mice [24]. Furthermore, the lack of PTX3 in a proatherogenic background may be associated with an increased inflammatory status in the vascular wall, which in turn contributes to the atherogenic process. In contrast, the transgenic overexpression of PTX3 has been found to result in greater resistance to lipopolysaccharide toxicity and cecal ligation and puncture [26]. There is also evidence that PTX3 may modulate inflammation-associated tissue damage.

PTX3 has also been found to offer protection against atherosclerosis. As a relationship between PTX3 and the cell adhesion molecule P-selectin in atherosclerotic lesions has recently been reported, it is possible that PTX3 may exert some of these effects through an association with this protein [27]. For instance, neutrophils rolling on P-selectin in venules at the sites of infection or injury receive signals that cause the release of PTX3 from specific granules. This released PTX3 then selectively binds locally expressed P-selectin, but not E- or L-selectin, in a paracrine manner, while the dissociation of this complex is slowed by the increased binding avidity due to the multimeric nature of PTX3. As more neutrophils roll, they release more PTX3, which then binds more P-selectin molecules. This constitutes a local negative feedback system that diminishes neutrophil tethering, accelerates rolling, and enhances detachment. Indeed, PTX3 expression has been found to decrease the number of neutrophils rolling on P-selectin *in vitro* in a concentration-dependent manner, while the injection of PTX3 *in vivo *has been shown to reduce the number of neutrophils rolling in thrombin-stimulated mesenteric venules of mice because PTX3 competitively inhibited between P-selectin and P-selectin glycoprotein 1 (PSGL-1) bonds.

The source of anti-inflammatory PTX3 has also been examined. By transplanting wild-type or PTX3-deficient bone marrow into irradiated wild-type or PTX3-deficient recipient mice, Deban et al. showed that PTX3 from hematopoietic cells is required to suppress neutrophil recruitment into the pleural cavity in the first 2 hours after chemokine challenge. In this short time frame, neutrophils are the likely source of PTX3, as they are the only hematopoietic cells that store PTX3 [28].

Very recently, Maugeri et al. have reported data that supports the release of PTX3 from activated neutrophils by platelets in patients with ACS [28]. In this study, the total amount of PTX3 in the neutrophils of patients with early AMI (early onset; <6 hr), late AMI (<48 hr), stable coronary artery disease, and healthy volunteers was measured using FACS. As found in our study, the maximum plasma level of PTX3 was reached at 6 hours after onset. Interestingly, the lowest PTX3 levels were found in the neutrophils of patients with early AMI, whereby confocal microscopy detected very low PTX3 expression in neutrophils from patients with early AMI and much higher PTX3 expression in neutrophils from patients with late AMI. Furthermore, released PTX3 from patients with early AMI was found to aggregate platelets expressing P-selectin compare with late AMI [28]. From these findings, PTX3 works as a cardioprotective to bind to activated circulating platelets and reduce the inflammation status in cardiovascular bed.

It has also been shown that plasma PTX3 levels increase significantly during widespread inflammations, such as sepsis [29]. In such scenarios, activated endothelial cells, dendritic cells, and/or macrophages may be major sources of PTX3, and although it has recently been demonstrated that PTX3 inhibits P-selectin-dependent adhesion [27], other, still undefined, mechanisms may also contribute to its anti-inflammatory properties *in vivo*.

### 3.3. Physiological PTX3 Levels

**Figure 1:**
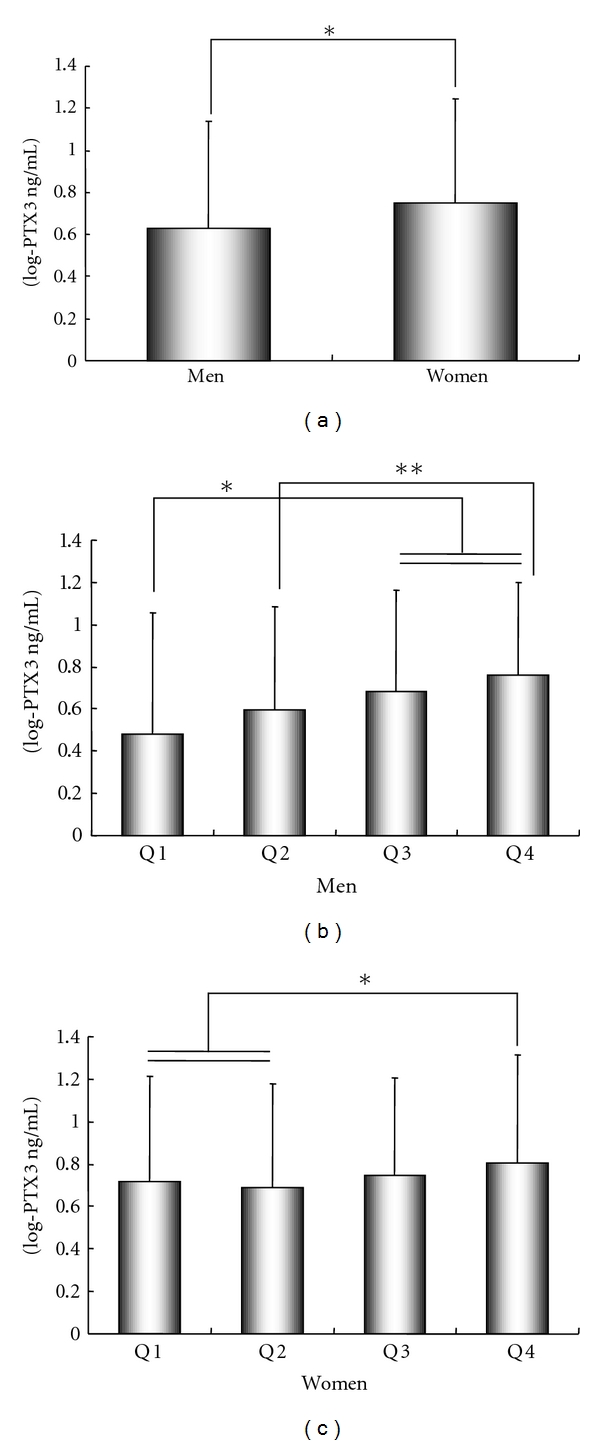
Geometric mean PTX3 plasma levels in men and women [25]. (a) Mean and confidence interval of natural log transformed PTX3 in men and women. Plasma PTX3 levels in men are significant lower than those in women (men, 1.87 (1.81, 1.94) ng/mL; women, 2.12 (2.05, 2.19) ng/mL). *P < 0.0001. (b) Plasma PTX3 levels according to quartiles of age in men. Quartile 1 (Q1): 37–49 years old; 1.62 (1.50, 1.74) ng/mL. Quartile 2 (Q2): 50–57 years old; 1.82 (1.70, 1.94) ng/mL. Quartile 3 (Q3): 58–68 years old; 1.98 (1.86, 2.11) ng/mL. Quartile 4 (Q4): 69–87 years old; 2.14 (2.02, 2.27) ng/mL. *P < 0.001, Q1 versus Q3 and Q4; **P < 0.0006, Q1 and Q2 versus Q4. (c) Plasma PTX3 levels according to quartiles of age in women. Quartile 1 (Q1): 38–52 years old; 2.05 (1.92, 2.18) ng/mL. Quartile 2 (Q2): 53–61 years old; 1.99 (1.87, 2.12) ng/mL. Quartile 3 (Q3): 62–70 years old; 2.10 (1.98, 2.23) ng/mL. Quartile 4 (Q4): 71–85 years old; 2.23 (2.02, 2.46) ng/mL. *P < 0.05, **P < 0.01.

The normal physiological concentration of plasma PTX3 expression has been determined to be approximately 2 ng/mL in a study that examined PTX3 levels in 1749 subjects (818 men and 931 women) [25]. Interestingly, plasma PTX3 levels were found to be significantly lower in men than in women (1.87 (1.81, 1.94) ng/mL versus 2.12 (2.05, 2.19) ng/mL, *P* < 0.0001) (Figure 1(a)). They were also found to be significantly higher in the oldest age group in both men and women (lowest quartile 1.62 (1.50, 1.74) ng/mL versus highest quartile 2.14 (2.02, 2.27) ng/mL in men, *P* < 0.001; lowest quartile 2.05 (1.92, 2.18) ng/mL versus highest quartile 2.23 (2.02, 2.46) ng/mL in women, *P* < 0.05; Figures 1(b) and 1(c)). PTX3 levels were also inversely correlated with triglyceride levels (*r* = −0.19 in men and *r* = −0.18 in women, *P* < 0.00001), and body mass index (*r* = −0.16 in men and *r* = −0.24 in women, *P* < 0.00001).

### 3.4. PTX3 Independence from Established Risk Factors

Plasma PTX3 levels have also been shown to be independent of other coronary risk factors, including total cholesterol, high-density lipoprotein (HDL) cholesterol, hemoglobin A1C, smoking status, gender, and obesity (Table 1) [17]. Although Yamashina et al. have reported a brachial-ankle pulse wave velocity (ba PWV) cutoff value of 14.0 m/s for screening subjects at risk of developing cardiovascular diseases in the general population [30], plasma PTX3 levels are not different between patients with ba PWV values of more or less than 14.0 m/s, or an intimal thickness of the carotid artery of more or less than 1.0 mm, which means within normal limits [17]. 

## 4. PTX3 in Other Diseases

The human PTX3 proximal promoters contain AP-1, NF-kappa B, Sp-1, and NF-IL6 binding sites [5]. Consequently, PTX3 is expressed in response to proinflammatory signals, including bacteria, IL-1 (but not IL-6), and TNF-alpha produced by primarily endothelial cells, neutrophils, and macrophages. As a result, inflammation diseases, especially disorders of the immune system such as rheumatoid arthritis [31], progressive systemic sclerosis [32], Chug-Straus syndrome, Wegener's granulomatosis, and microscopic polyangiitis [33], as well as systemic inflammatory response syndrome (SIRS) [29, 34], result in increased expression of plasma PTX3. Chronic kidney disease is also known to increase the level of plasma PTX3 [35, 36]. Therefore, it was also of interest to determine the PTX3 expression patterns in inflammatory bowel diseases such as Crohn's disease and ulcerative colitis. As IL-6 was found to have increased expression in active Crohn's disease, but not in ulcerative colitis, it is not surprising that plasma PTX3 levels were increased in patients with only ulcerative colitis (because IL-1, but not IL-6, causes induction of PTX3 expression). PTX3 may therefore also be a good diagnostic marker for deterioration in patients with inflammatory bowel disease [37, 38].

## 5. Conclusion

Advances in genomics and proteomics technologies have led to the discovery of many novel biomarkers that provide valuable information, which can be used in disease screening and diagnosis, determining prognoses, and therapeutic monitoring. One potentially useful biomarker for cardiovascular disease is PTX3, and many studies have recently examined this protein in clinical situations. Although PTX3 is in the same protein family as CRP, it is expressed predominantly in atherosclerotic lesions. Interestingly, the expression of PTX3 in endothelial cells has been shown *in vitro* to be suppressed to a greater extent by pitavastatin than other genes. We have therefore recently determined the normal physiological concentration of PTX3. As PTX3 has promise as a biomarker for cardiovascular disease, we have recently determined the normal physiological concentration of this protein. In addition, kits capable of detecting PTX3 are available, including a highly sensitive kit recently developed by our group, facilitating the use of PTX3 as a biomarker. Additional clinical study will be necessary to further elucidate the role of this protein in cardiovascular disease.

## Figures and Tables

**Table 1:** Geometric mean PTX3 plasma levels by CHD risk factors [17].

Risk factor		PTX3 (ng/mL; 95% CI)	*P* value
TCHO	≧220 mg/dL	2.16 (1.85–2.46)	0.51
	<220 mg/dL	2.30 (2.01–2.60)	
HDL	≧40 mg/dL	2.23 (1.98–2.48)	0.42
	<40 mg/dL	2.03 (1.62–2.43)	
HgbA1C	≧5.9%	2.12 (1.83–2.42)	0.29
	<5.9%	2.36 (2.02–2.66)	
Obesity	≧24.2 kg/m^2^	1.98 (1.64–2.32)	0.07
	<24.2 kg/m^2^	2.39 (2.12–2.67)	
IMT	≧1.0 mm	2.30 (2.02–2.58)	0.56
	<1.0 mm	2.24 (1.80–2.68)	
Smoke	Smoking	2.32 (1.96–2.68)	0.58
	None	2.20 (1.93–2.47)	
Gender	Male	2.26 (1.97–2.54)	0.84
	Female	2.22 (1.93–2.51)	

CHD: coronary heart disease.

TCHO: total cholesterol; HDL: high-density lipoprotein; HgbA1C: hemoglobin A1C; IMT: intimal media thickness.

CI: confidence interval.
